# Supplementary figures and images for: Neuronal Constituents and Putative Interactions Within the Drosophila Ellipsoid Body Neuropil
Source: Front Neural Circuits. 2018 Nov 27;12:103. doi: 10.3389/fncir.2018.00103 (PMC6278638; doi:10.3389/fncir.2018.00103)

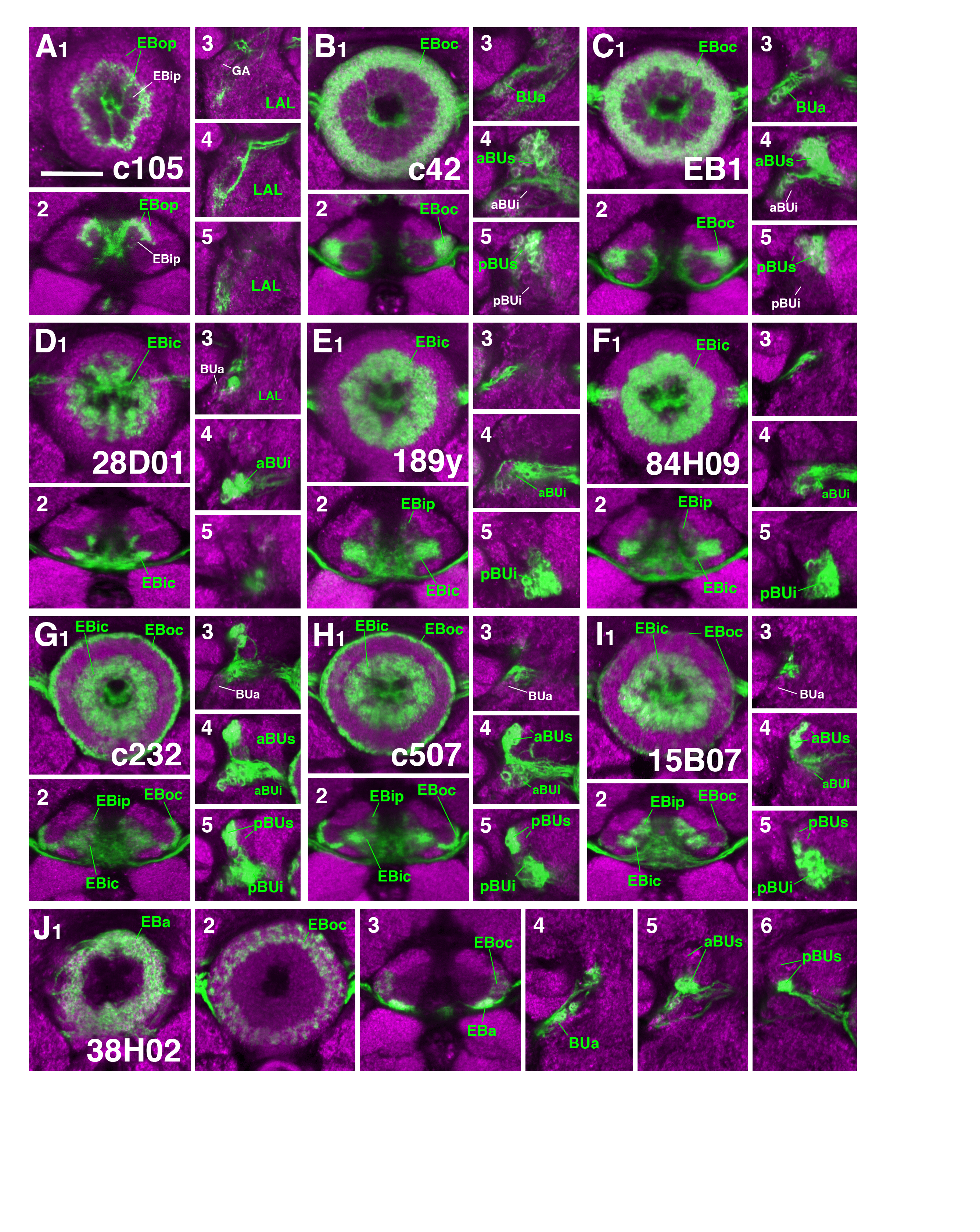

Supplement: Supplementary file 8 [file Image_1.JPEG]

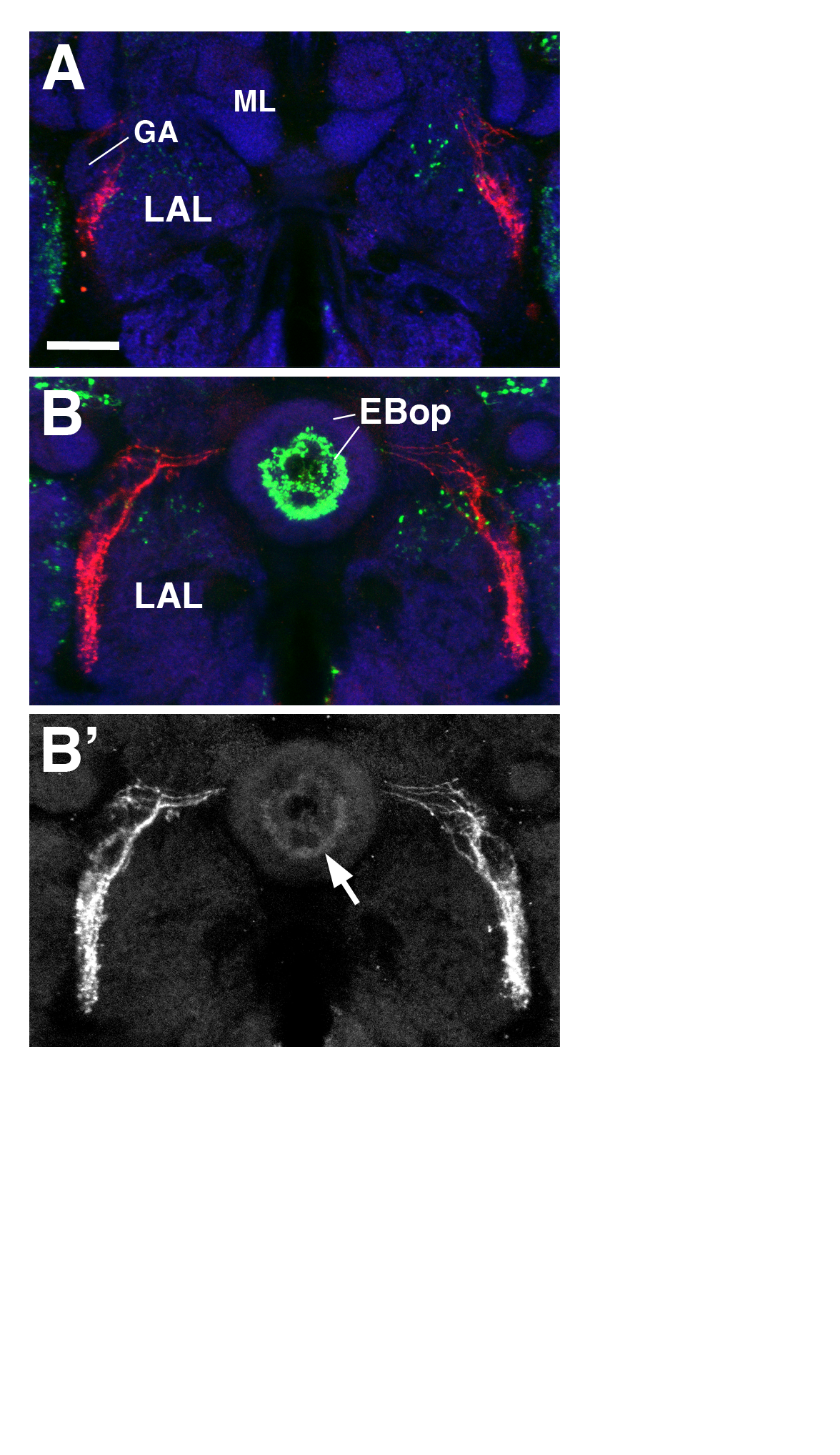

Supplement: Supplementary file 9 [file Image_2.TIF]
