# Supplementary material for: Neuronal Constituents and Putative Interactions Within the Drosophila Ellipsoid Body Neuropil
Source: Front Neural Circuits. 2018 Nov 27;12:103. doi: 10.3389/fncir.2018.00103 (PMC6278638; doi:10.3389/fncir.2018.00103)
Supplement: Supplementary file 10 [file Table_1.docx]

**Supplemental Figure Legends**

**Supplemental Figure 1**

R-neuron subclasses labeled by Gal4 drivers from previous studies

(A-I) Confocal z-projections of Gal4 drivers that label R-neuron subclasses. Each lettered, five-paneled module corresponds to an individual driver labeled with 10xUAS-mCD8::GFP. Within each module, GFP-labeled neurons are shown in green; neuropil is labeled with anti-DN-cadherin (magenta). Top left (1) is a high magnification, frontal view of the ellipsoid body at an antero-posterior level (anterior, intermediate, or posterior) that highlights the circular arbor of a given driver most clearly. Bottom left (2) is a horizontal section visualizing all five DN-cadherin positive domains. Second column (3-5) are three frontal sections of the bulb at different antero-posterior depths (as described in Fig.1); from top to bottom: anterior section containing the anterior bulb (BUa), intermediate section containing the anterior regions of the superior (aBUs) and inferior (aBUi) bulb, posterior section containing the posterior regions of the superior (pBUs) and inferior (pBUi) bulb. R1 innervates the lateral accessory lobe (LAL) rather than the bulb, in which case the same sections are shown at a more ventral position. Highlighted in large green text is the domain predominantly innervated by the R-neuron subclass; smaller green text signifies additional regions of innervation. Small white text in all panels denotes relevant spatial landmarks.

(A1-5) c105-Gal4 (R1). c105-positive neurons project from the LAL to EBop in a pattern characteristic of R1 (compare to Fig.2A). (A1) Posterior EB section.

(B1-5) c42-Gal4 (R2 + R4m). c42-Gal4-positive neurons project from BUs to EBoc in a pattern characteristic of R2 (compare to Fig.2B). In addition, microglomeruli in BUa are labeled (B3), suggesting that R4m neurons are also present (compare to Fig.3B). (B1) Intermediate EB section.

(C1-5) EB1-Gal4 (R2 + R4m). EB1-Gal4-positive neurons exhibit the same projection pattern as that of c42, suggesting the presence of R2 and R4m (compare to Supp. Fig.1B1-5). (C1) Intermediate EB section.

(D1-5) R28D01-Gal4 (R3m). R28D01-Gal4-positive neurons project from BUi (aBUi in particular; D4) to EBic in a pattern characteristic of R3m (compare to Fig.2D). (D1) Anterior EB section.

(E1-5) 189y-Gal4 (R3d + R3p). 189y-Gal4-positive neurons project from BUi to EBic in a pattern characteristic of R3d (compare to Fig.2C). In addition, EBip is also labeled, suggesting that R3p neurons are also present (compare to Fig.2F). (E1) Intermediate EB section. As multiple drivers exhibit this pattern (see Supp. Fig.1F-I below), 189y may also label a unique subclass that individually branches in both domains.

(F1-5) R84H09-Gal4 (R3d + R3p). R84H09-Gal4-positive neurons exhibit the same projection pattern as that of 189y (compare to Supp. Fig.1E1-5), suggesting the presence of R3d and R3p. (F1) Intermediate EB section.

(G1-5) c232-Gal4 (R3d + R3p + R4d). c232-Gal4-positive neurons exhibit the same projection pattern as that of 189y (compare to Supp. Fig.1E1-5), suggesting the presence of R3d and R3p. In addition, c232-positive neurons project from BUs to EBoc in a pattern characteristic of R4d (compare to Fig.3A). (G1) Intermediate EB section.

(H1-5) c507-Gal4 (R3d + R3p + R4d). c507-Gal4-positive neurons exhibit the same projection pattern as that of c232, suggesting the presence of R3d and R3p, as well as R4d (compare to Supp. Fig.1G1-5). (H1) Intermediate EB section.

(I1-5) R15B07-Gal4 (R3d + R3p + R4d). R15B07-Gal4-positive neurons exhibit the same projection pattern as that of c232, suggesting the presence of R3d and R3p, as well as R4d (compare to Supp. Fig.1G1-5). (H1) Intermediate EB section.

(J1-6) Confocal z-projections of R38H02-Gal4 (R5 + R4m). R38H02-Gal4 contains six panels due to the presence of distinct neuron types represented across multiple sections. As in A-I, 10xUAS-mCD8::GFP-labeled neurons are shown in green; neuropil is labeled with anti-DN-cadherin (magenta). Left two panels (1 and 2) are high magnification, frontal views (anterior and intermediate sections) of the ellipsoid body. (3) is a horizontal section visualizing all five DN-cadherin positive domains. (4-6) are three frontal sections of the bulb at different antero-posterior depths as in other modules. R38H02-positive neurons project from BUs to EBa in a pattern characteristic of R5 (compare to Fig.3C). In addition, (J4) microglomeruli in BUa and (J2-3) EBoc innervation pattern is visible and suggesting that R4m neurons are also present (compare to Fig.3B).

Other abbreviations: GA, gall.

Scale bar represents 25 µm (A_1-2_-J_1-2_; J_3_).

**Supplemental Figure 2**

R1-neurons are predominantly dendritic in the lateral accessory lobe and axonal in the ellipsoid body

(A,B,B’) Confocal z-projections of R31A12-Gal4 (R1). (A-B) R1-neurons labeled with the presynaptic marker syt.EGFP (green) and dendritic marker DenMark (red). Neuropil labeled by anti-DN-cadherin (blue). (B’) shows the isolated DenMark signal from (B) in gray, demonstrating strong dendritic labeling in the lateral accessory lobe (LAL) and weaker, but visible expression in the outer posterior domain of the ellipsoid body (EBop; arrow). Larger white annotations denote arborization-containing domains of interest; smaller white annotations represent spatial landmarks.

Other abbreviations: GA, gall; ML, medial lobe of the mushroom body.

Scale bar represents 25 µm (A-B’).

**Supplemental Movies Legends**

Confocal z-stacks of Gal4 drivers that label distinct ring neuron subclasses. Each Gal4 line is represented by a pair of movies, in which the driver line is labeled with 10xUAS-mCD8::GFP (green) and the neuropil is labeled with anti-DN-cadherin (magenta). Odd numbered movies and even numbered movies progress from anterior to posterior (AP) sections and dorsal to ventral (DV) sections, respectively. Each movie begins with the ring neuron designation and its associated driver line. The movies pause and text/hatched lines appear in conjunction with significant landmarks. Text and hatched lines are denoted in green if they contain notable portions of the ring neurons; other landmarks/text are denoted in white. AP movies contain the following texts/landmarks: Cell bodies; Gall of the lateral accessory lobe (Gall of LAL); lateral accessory lobe (LAL); anterior bulb (BUa), anterior region of the superior (aBUs) and inferior (aBUi) bulb, posterior region of the superior (pBUs) and inferior (pBUs) bulb; anterior (EBa), inner central (EBic), outer central (EBoc), inner posterior (EBip), and outer posterior (EBop) domains of the ellipsoid body. 14G09 contains additional blue designations for prominent innervation by the driver line that are likely due to other neurons besides ExR4 (posterior part of the LAL, Noduli, and Fan-shaped body). DV movies only contain EB domain designations; each domain is initially labeled in white, followed by the individual domain with the most prominent innervation in green.

Supplemental Movies 1 and 2 (31A12-Gal4; R1).

Supplemental Movies 3 and 4 (78B06-Gal4; R2).

Supplemental Movies 5 and 6 (80C07-Gal4; R3d).

Supplemental Movies 7 and 8 (28E01-Gal4; R3m).

Supplemental Movies 9 and 10 (12G08-Gal4; R3a).

Supplemental Movies 11 and 12 (VT063949-Gal4; R3p).

Supplemental Movies 13 and 14 (VT057232-Gal4; R3w).

Supplemental Movies 15 and 16 (12B01-Gal4; R4d).

Supplemental Movies 17 and 18 (59B10-Gal4; R4m).

Supplemental Movies 19 and 20 (58H05-Gal4; R5).

Supplemental Movies 21 and 22 (VT011965-Gal4; R6).

Supplemental Movies 23 and 24 (14G09-Gal4; ExR4).
